# The Pros & Cons of Measuring Plasma Ammonia in the Management of Patients With Cirrhosis: An ISHEN Debate

**DOI:** 10.1111/liv.70904

**Published:** 2026-10-22

**Authors:** Maria Pilar Ballester, Patricia Bloom, Christopher F. Rose

**Affiliations:** ^1^ Digestive Disease Department Clinic University Hospital of Valencia, INCLIVA Biomedical Research Institute Valencia Spain; ^2^ Department of Medicine Mayo Clinic Rochester Minnesota USA; ^3^ Hepato‐Neuro Laboratory, Department of Medicine Université de Montréal, Centre de Recherche du Centre Hospitalier de L'université de Montréal (CRCHUM) Montreal Quebec Canada

## Abstract

Ammonia is a nitrogenous molecule which is primarily generated in the gastrointestinal tract. The neurotoxicity of ammonia plays a critical role in the liver's regulation of circulating ammonia levels. In liver disease, an increase in blood ammonia has been long implicated in the pathogenesis of hepatic encephalopathy (HE), a neurological complication of liver disease. However, the clinical value of measuring plasma ammonia in patients with cirrhosis remains controversial. This dilemma was the subject of a debate held at the International Society of Hepatic Encephalopathy and Nitrogen Metabolism (ISHEN) meeting in St‐Paulin, Québec, Canada in October 2025. This review illustrates the pros & cons of measuring plasma ammonia in the management of patients with cirrhosis.

## Key Points

1. Ammonia is an important factor in hepatic encephalopathy; however, ammonia is not the sole factor causing encephalopathy during cirrhosis.
2. Plasma ammonia in cirrhosis is not routinely measured since the levels of ammonia may not guide treatment.
3. High ammonia levels are associated with complications of cirrhosis, including hepatic encephalopathy, but it remains unknown whether routinely measuring or lowering ammonia improves patient outcomes.
4. Internationally standardized methods are needed to make ammonia measurements more reliable and comparable between hospitals and research centres.

## Lay Summary

Measuring blood ammonia in patients with cirrhosis remains controversial, as it may help predict hepatic encephalopathy (HE) and other complications, assess risk and monitor responses to ammonia‐lowering treatments. However, ammonia levels often do not influence clinical decisions, and unreliable measurements may contribute to unnecessary treatment and adverse effects. Standardized testing protocols and clinical trials are needed to determine whether measuring or lowering ammonia improves patient outcomes and enables more personalized care.

## 1 QUESTION: Is It Useful to Measure Plasma Ammonia Levels in a Patient With Cirrhosis in Routine Clinical Practice?

### 1.1 Background on Ammonia

Over a century ago, the first observations were made suggesting a link between elevated blood ammonia and brain dysfunction. In 1893, a portacaval shunt was placed in dogs, a surgical procedure first described by Eck in 1879 (‘Eck's fistula’) [1] and within 10–42 days following surgery, neurobehavioral changes (aggression, irritability, ataxia, convulsions and coma) developed. Interestingly, these symptoms worsened following ingestion of meat which led to the term ‘meat intoxication syndrome’. Later this syndrome was associated with an increase in ammonium salts in the urine [2]. In another experiment, following ingestion of ammonia, dogs fell into a coma and died. The brain ammonia content was four times higher than normal dogs. It was then concluded the observed neurological dysfunction which occurred in these dogs was a result of ammonia not being able to be converted to urea in the liver, though admittedly this was not an animal model of chronic liver disease [3].

Ammonia is primarily generated within the gastrointestinal tract where bacteria digest ingested proteins, deaminate amino acids and hydrolyze urea, releasing ammonia as a by‐product. In addition, enterocytes, the columnar epithelial cells lining the inner surface of the small and large intestines, contain the enzyme glutaminase, which catalyzes the deamination of glutamine to glutamate and ammonia.

The liver plays a central role in regulating blood ammonia concentrations through the complementary metabolic functions of periportal and perivenous hepatocytes. Periportal hepatocytes express the enzymes of the urea cycle, which convert ammonia derived from the portal circulation into non‐toxic urea. Any ammonia that escapes periportal detoxification is subsequently removed by perivenous hepatocytes, which express glutamine synthetase (an enzyme which catalyzes the amination of glutamate and ammonia to glutamine). Liver failure, liver disease, urea cycle disorders as well glutamine synthetase deficiency undeniably lead to an increased risk of hyperammonemia.

Decades of studies have demonstrated the deleterious effects of ammonia on the brain and as a result, ammonia has been implicated as a pathogenic factor in the pathophysiology of hepatic encephalopathy (HE), a complex neurological complication of liver failure and disease [4].

In aqueous solutions, ammonia exists as a dissolved gas (NH_3_) and as an ion (NH_4_^+^), with the ratio of NH_3_ to NH_4_^+^ being pH‐dependant. At pH 7.4, close to 2% is represented as NH_3_ with 98% existing as NH_4_^+^. Following their respective concentration gradients, NH_3_ freely diffuses across plasma membranes while NH_4_^+^ is transported into and out of cells through aquaporins and ammonia transporters. In addition, since the characteristics of the NH_4_^+^ ion are very similar to the K^+^ ion (similar ionic radius and diffusion coefficient), NH_4_^+^ can cross plasma membranes via K^+^ channels and co‐transporters [5]. This characteristic feature of NH_4_^+^ is particular to ammonia since it is not found in other weak bases with similar pKa. Therefore, the similarities between NH_4_^+^ and K^+^, conclude that elevated ammonia impacts cellular membrane potential. In addition, based on the fact that NH_3_ can accept a proton and NH_4_^+^ can donate a proton, an increased flux of ammonia has a direct effect on the pH of the environment (intracellular and extracellular). Lastly, ammonia is utilized and generated in multiple biochemical reactions and therefore an increased influx of ammonia will inadvertently lead to alterations in metabolism [5]. Within the brain, the astrocyte bears the brunt in attempting to detoxify toxic levels of ammonia since astrocytes specifically express glutamine synthetase. Consequently, an accumulation of intracellular astrocytic glutamine leads to osmotic imbalance and cellular oedema [6]. For this, elevated brain ammonia is the primary pathogenic factor involved in the development of brain oedema [7]. In addition, the direct effects of high ammonia can consequently lead to a cascade of pathophysiological mechanisms, exerting deleterious effects through multiple pathways including inflammation, oxidative stress, mitochondrial dysfunction and disruption of cellular bioenergetics [5].

The role and value of measuring ammonia for the management of patients with cirrhosis was debated during the ISHEN meeting in St‐Paulin Québec, Canada in October 2025. The following review summarizes the arguments presented by both sides during the debate (Figure 1). The views described do not represent the positions of all listed authors.

**FIGURE 1:**
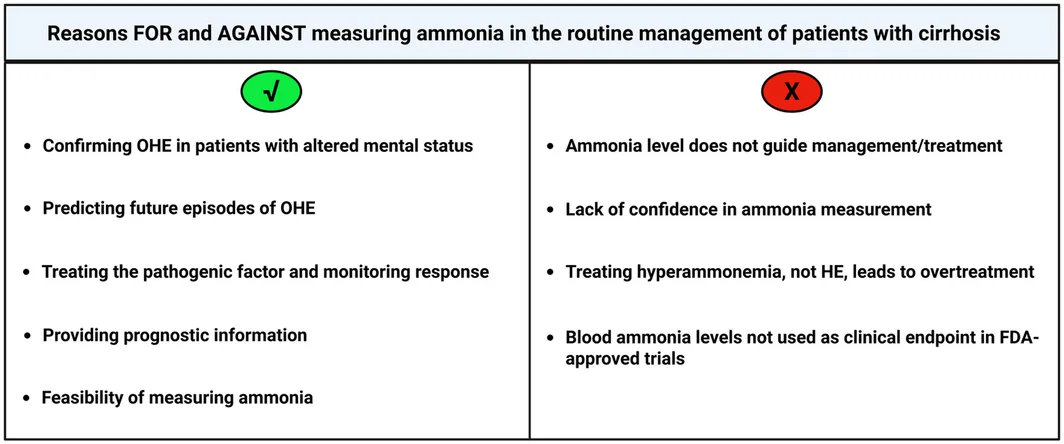
Reasons for and against measuring ammonia in the routine management of patients with cirrhosis. FDA, food and drug administration; HE, hepatic encephalopathy; OHE, overt hepatic encephalopathy.

## 2 Should Plasma Ammonia Be Measured in the Routine Management of Patients With Cirrhosis? YES, For the Following Reasons

### 2.1 Defining Hepatic Encephalopathy in Patients With Altered Mental Status

In patients with clinical symptoms of encephalopathy, blood ammonia measurement can be useful in confirming the diagnosis of HE as a normal ammonia value would suggest other causes of encephalopathy (associated with liver disease) or differential diagnosis (unrelated to liver disease). A recent retrospective study including 530 patients hospitalized with an acute decompensation, patients were categorized into four groups based on the presence of encephalopathy and ammonia levels. The authors showed that 33% of patients with acute overt encephalopathy did not associate with high ammonia levels. In this cohort, defined as non‐HE, encephalopathy was associated with significantly more medications and a higher occurrence of infections compared to patients with overt HE (OHE) [8]. As a result, this cohort received a higher rate of antibiotics. Therefore, measuring plasma ammonia can confirm the diagnosis of OHE and prevent the unnecessary administration of lactulose.

### 2.2 Predicting Future Episodes of Overt Hepatic Encephalopathy and Other Liver‐Related Complications

Emerging evidence supports the utility of measuring ammonia for risk stratification in clinically stable outpatients with cirrhosis. A prospective multicentre European study of 754 outpatients with cirrhosis demonstrated that ammonia is an independent predictor of hospitalization with liver‐related complications, including OHE, variceal bleeding, ascites, bacterial infection and mortality, with a higher predictive accuracy than traditional severity scores such as the MELD score [9]. Three additional studies, two from Europe and one from Asia reproduced the utility of ammonia as a significant risk factor for hospitalization due to liver‐related complications and death [10, 11, 12]. Similarly, four additional studies which included the performance of neuropsychometric tests demonstrated ammonia as an independent predictor of OHE [12, 13, 14, 15].

In addition, two recent studies developed prognostic models incorporating ammonia levels. One of the models replaced HE with ammonia in the Child‐Pugh score showing a good predictive performance for acute decompensation [16]. The second model, called the AMMON‐OHE model, was developed to predict a first episode of OHE and included ammonia, sex, diabetes, albumin and creatinine. This model outperformed neuropsychometric tests, Child‐Pugh score and their combinations with other clinical and biochemical variables to predict OHE [12] and more recently, predicted other liver‐related complications [17].

Furthermore, in the context of patients undergoing TIPS, several studies have demonstrated the utility of ammonia to predict post‐TIPS outcomes. In two prospective and three retrospective studies, baseline ammonia levels (pre‐TIPS) demonstrated to be an independent risk factor for post‐TIPS OHE [18, 19, 20, 21, 22]. Two of these studies developed predictive models. The first one included ammonia levels, age, indocyanine green retention rate at 15 min, percentage reduction in portal pressure gradient and creatinine, and outperformed the Child‐Pugh and MELD scores in predicting OHE in both the training and validation cohorts [21]. The second one incorporated pre‐TIPS blood ammonia, MELD score, notable hydrothorax, alcohol‐related cirrhosis, stent diameter and the decrement in portal pressure gradient and showed a higher accuracy than any individual risk factor alone to predict post‐TIPS OHE [20]. A recent retrospective study demonstrated that the AMMON‐OHE model outperforms liver function scores in predicting post‐TIPS OHE, with application of the model at 1 month after the procedure performing the best [23]. Similarly, the modified Child‐Pugh score based on plasma ammonia levels outperformed other risk scores such as Child‐Pugh, MELD, MELD‐Na and ALBI scores in predicting post‐TIPS transplant‐free survival [24].

Collectively, these findings exemplify the value of measuring ammonia in order to identify patients at high risk of OHE and other liver‐related complications, facilitating early interventions and personalized management strategies.

### 2.3 Treating the Pathogenic Factor and Monitoring Response in Patients With Encephalopathy

Specific treatment strategies for HE aims to reduce ammonia levels by either decreasing ammonia production or increasing its elimination. Lactulose and rifaximin are the first‐line treatment for patients with HE and are expected to reduce ammonia absorption by enhancing colonic excretion and suppressing urease‐producing bacteria, respectively [25, 26]. However, in routine clinical practice, patients are often not categorized by ammonia levels and ammonia analyses are not systematically performed, which can lead to overtreatment of patients that do not really benefit from ammonia‐lowering drugs. In a phase 2b study of ornithine phenylacetate which included hospitalized patients with cirrhosis and OHE with hyperammonemia, HE improvement was directly associated with ammonia reduction from baseline and no beneficial effect was observed in patients without a decrease in plasma ammonia [27]. Interestingly, patients in the control group (standard care with lactulose and/or rifaximin) did not present a significant reduction in blood ammonia from baseline [27]. A prospective longitudinal study of patients admitted to the intensive care unit (ICU) demonstrated that improvement in OHE was associated with a reduction in ammonia levels compared to those that did not improve which was associated with an increased risk of death [28]. Similarly, two longitudinal studies of hospitalized patients with acute decompensation or acute‐on‐chronic liver failure (ACLF) showed that a decrease in ammonia levels on days 4–7 compared to baseline was associated with improvement or no change in HE grades, while an increase in ammonia was associated with worsening HE [29, 30]. This evidence suggests that ammonia should be considered a companion biomarker to select patients for treatment with an ammonia lowering drug and to monitor response and change treatment in patients who do not improve HE.

### 2.4 Providing Prognostic Information in Patients With Acute Decompensation or Acute‐On‐Chronic Liver Failure

Blood ammonia measurement can be useful in the management of hospitalized patients with cirrhosis, particularly in those presenting with acute decompensation or ACLF. Several studies demonstrated that ammonia provides prognostic information regarding risk of organ failures and death. Specifically, one US‐single centre retrospective study of 494 patients and two prospective studies of 498 and 701 acutely decompensated or ACLF patients from India and the UK, and from Asia, respectively, demonstrated that ammonia levels predict 28‐day mortality [31, 32, 33], regardless of the presence of OHE. Two additional studies of patients with ACLF or admitted in the ICU, respectively, also showed that ammonia levels predict in‐hospital mortality, again, regardless of the presence of OHE [34, 35]. In the latter study, the authors demonstrated that serial ammonia measurement between Days 1 and 3 adds predictive information regarding risk of in‐hospital mortality, with a good discrimination and calibration in a validation cohort of 600 patients.

### 2.5 Feasibility of Ammonia Measurement

The measurement of ammonia is inexpensive and widely available in all clinical laboratories. The measurement of ammonia provides a quick and objective biomarker, easily reproducible when measured under recommended conditions. In addition, the description of ammonia levels as a function of the upper limit of normal (ULN) for the reference laboratory serves to normalize ammonia levels making comparison across different hospitals better interpreted [9, 10, 36].

## 3 Should Plasma Ammonia Be Measured in the Routine Management of Patients With Cirrhosis? NO, For the Following Reasons

### 3.1 Ammonia Level Does Not Influence Management/Treatment of Patients With OHE

In a study of 1202 patients with cirrhosis admitted with OHE, 46% had an ammonia level drawn on admission from which 60% had hyperammonemia [37]. Patients with ammonia levels drawn were similar in clinical characteristics to those without an ammonia level drawn. There was no relationship between admission ammonia level and the volume of lactulose the patient received in the first 48 h of hospitalization. In other words, the ammonia level did not influence treatment the patient received. Furthermore, there was no difference in lactulose dose between patients who had ammonia levels drawn versus those who did not. In the same study, there was also no difference in the time to OHE resolution between the patients with and without ammonia checked and also between the patients with high or normal ammonia levels. The authors furthermore conducted propensity score matching and found no difference in lactulose dosing between those with or without ammonia levels drawn. Rifaximin treatment was also not impacted by blood ammonia levels. Finally, there was no impact of ammonia levels on other important clinic outcomes like overall length of stay, ICU needs, ICU length of stay or inpatient mortality. This real world study demonstrated that there is no practical impact of checking plasma ammonia levels since they did not impact treatment or outcomes.

In an another real‐world study, authors called healthcare providers immediately after they ordered an ammonia level [38]. They asked about pre‐test probability and post‐test probability of OHE. They found that when controlling for pre‐test probability, ammonia level did not influence the final diagnosis or whether or not the patient received lactulose. In other words, HE is a clinical diagnosis and is not assisted by an ammonia level.

### 3.2 Lack of Confidence in Ammonia Measurement

Ammonia is unstable and is easily affected during many pre‐analytical steps and ammonia measurement lacks standardized protocols across hospital laboratories. Many common factors can influence reported ammonia levels such as sampling site (arterial vs. venous), type of tube used (heparin, EDTA coated) for collection, with or without a tourniquet, fist clenching and hemolysis. In addition, sample handling is also critical; temperature (placing blood sample on ice), time to centrifugation (spin down cells), transportation to laboratory (with or without ice) and time before sample is analyzed can all impact the results [39, 40, 41]. In addition, variations in assay methodology and laboratory protocols can influence the quantification of blood ammonia. Collectively, each step could lead to falsely elevated ammonia levels due to ongoing cellular metabolism. As a result, these issues make ammonia one of the most labile lab tests. Therefore, even though a normal ammonia is accepted with confidence, false positives are a concern. Moreover, different assays, as well as different analysers are used between clinical hospital laboratories resulting in various ranges [42]. Therefore, a lack standardization across institutions remains unmet.

### 3.3 Treating Hyperammonemia Without Confirmed Diagnosis of OHE Leads to Overtreatment

Measuring blood ammonia without a confirmed diagnosis of OHE can lead to needlessly starting a patient on lactulose or rifaximin. Overuse of lactulose can lead to diarrhoea, abdominal cramping, bloating and flatulence as well as dehydration and electrolyte disturbances, including hypernatremia [43]. Whereas inappropriate use of rifaximin can lead to antimicrobial resistance and long‐term use has been associated with an increased risk of sepsis and spontaneous bacterial peritonitis [44].

### 3.4 Ammonia Not Assessed in the FDA Approved Clinical Trial for the Treatment for HE

In the clinical trial which lead to the FDA‐approved treatment for HE, it was proven the recurrence of HE was significantly reduced in patients receiving rifaximin as add‐on therapy to lactulose [45]. This study included patients who were discharged following their second episode of OHE which were randomized to receiving lactulose or lactulose plus rifaximin and number of patients hospitalized with an HE‐related event was significantly reduced in the rifaximin (+lactulose) group. Importantly, ammonia was never measured throughout the study. Therefore, rifaximin is approved for treatment use in patients with recurrent HE, with the indication based on prior episodes and not on ammonia levels. In addition, lactulose is also indicated for the treatment and prevention of OHE in patients with cirrhosis, which does not require or depend on blood ammonia levels. Therefore, based on clinical trial data, ammonia levels are generally not used as a primary endpoint or a mandatory, consistent marker of treatment efficacy for OHE.

## 4 Conclusion

Measuring ammonia in patients with cirrhosis remains a dispute and there are strong arguments for why ammonia should be measured and for why ammonia should not be measured. In order to settle this debate, future studies need to be conducted. Similarly, to increase confidence in ammonia measurement, rigorous international standardization of pre‐analytical and analytical protocols should be adopted across institutions to ensure reproducibility and facilitate comparison of results across healthcare centres and research laboratories. In addition, single spot ammonia measurements may be suboptimal because of substantial intra‐day variability and their dependence on factors such as fasting status and muscle activity. The development of a reliable point‐of‐care ammonia assay could address these limitations by enabling rapid, standardized and serial measurements, thereby improving diagnostic accuracy, treatment monitoring and clinical decision‐making. As of today, a blood ammonia level will not guide treatment and therefore ammonia is not routinely measured. Lactulose and rifaximin are approved treatments for HE and are used independent of the concentration of plasma ammonia. There is a lot of evidence describing the important prognostic role of ammonia as well as its value in predicting future episodes of OHE. However, a clinical trial investigating whether attenuating or reducing blood ammonia levels leads to better outcomes has yet to be carried out. In addition, an approved ammonia‐lowering strategy would help drive these highly warranted clinical trials and conclude the value of measuring ammonia in clinical practice. The heterogeneity of the underlying pathogenic factors causing encephalopathy merits further validation and defining whether ‘pure’ HE is the diagnosis of encephalopathy and hyperammonemia. In turn, an FDA‐approved ammonia‐lowering strategy for the treatment of HE would justify measuring ammonia in clinical practice which would provide a treatment option to target the pathogenic factor and ultimately lead to improved response to treatment and better management with personalized medicine.

## Author Contributions

Maria Pilar Ballester (presented the PROS during the debate at the ISHEN 2025 meeting): conceptualization, writing, review and editing. Patricia Bloom (presented the CONS during the debate at the ISHEN 2025 meeting): conceptualization, writing, review and editing. Christopher F. Rose (president and organizer of the ISHEN 2025 meeting): conceptualization, writing, review and editing.

## Funding

Maria Pilar Ballester is supported by the Juan Rodés contract (JR23/00029), Instituto de Salud Carlos III (Spain).

## Conflicts of Interest

Maria Pilar Ballester has no conflicts of interest to declare. Patricia Bloom declares consultancy roles with Nexilico and Boehringer Ingelheim and has received research grants from Boehringer Ingelheim and Vedanta Biosciences. Christopher F. Rose declares consultancy roles with Genfit and has received research grants from Ardelyx, Genfit and Satellite Bio.

## Data Availability

Data sharing not applicable to this article as no datasets were generated or analysed during the current study.
